# Reducing medication errors in HIV-positive patients: Influence of a clinical pharmacist

**DOI:** 10.4102/sajhivmed.v25i1.1594

**Published:** 2024-08-01

**Authors:** Elmien Bronkhorst, Michè Joseph-Busby, Selente Bezuidenhout

**Affiliations:** 1 Department of Clinical Pharmacy, Sefako Makgatho Health Sciences University, Pretoria, South Africa; 2 Department of Public Health and Pharmacy Management, Sefako Makgatho Health Sciences University, Pretoria, South Africa

**Keywords:** HIV, opportunistic infections, people living with HIV, medication errors, co-morbidities, clinical pharmacist

## Abstract

**Background:**

The roll-out of antiretroviral medicines has improved life expectancy in people living with HIV (PLHIV). This has resulted in more patients being hospitalised for non-communicable diseases, increasing risk for medication errors (MEs). Pharmacists, through medication reconciliation, may identify and reduce MEs in this population.

**Objectives:**

To describe the importance of a pharmacist’s involvement in identifying and quantifying types of MEs.

**Method:**

A quantitative, prospective observational study was conducted over 14 weeks. A pharmacist reviewed HIV-positive, hospitalised patients’ files, using a data collection instrument, to determine the prevalence of MEs in PLHIV. The study pharmacist recommended appropriate actions to the prescriber to resolve MEs and documented resolution of the MEs.

**Results:**

The study population of *n* = 180 patient files were reviewed 453 times, identifying 466 MEs. Medication errors included incorrect medication reconciliation from history (19; 4.1%), prescription omission (17; 3.7%), duplication of therapy (10; 2.2%), missed doses (265; 57.1%), incorrect dosing (103; 22.2%), incorrect administration frequency (2; 0.4%), incorrect duration of therapy (15; 3.2%) and drug-drug interactions (18; 3.9%). More than half (58.2%) of the MEs were resolved in less than 24 h, with involvement of the pharmacist.

**Conclusion:**

This study demonstrates the magnitude of MEs experienced in hospitalised PLHIV and highlights the role clinical pharmacists play in identifying and resolving MEs to improve patient outcomes.

**What this study adds:** Medication monitoring in PLHIV by a clinical pharmacist significantly reduced the risk of medication errors a hospital setting.

## Introduction

Sub-Saharan Africa bears the largest global burden of the HIV pandemic (20.8 million),^1,2,3^ and South Africa (SA) is the most heavily affected country with an estimated 7.8m people living with HIV (PLHIV).^4,5^ In 2022, 37.7m PLHIV were known to have contracted the disease globally, with 74% having access to antiretroviral therapy (ART).^2^ SA has the most extensive ART programme globally, with approximately 83% of PLHIV in SA on ART in 2022.^2^ The availability of ART has transformed a fatal disease into a manageable chronic disease, increasing life expectancy amongst PLHIV. Concomitantly, an increase in life expectancy leads to increased co-morbidities. Up to a 45% increase in medication use, for treatment regimens for co-morbidities, results in increased risk of medication errors (MEs).^6,7,8,9,10^ Pharmacists can contribute by identifying MEs, preventing adverse drug reactions (ADEs), optimising ART, identifying potential drug-drug interactions, and managing co-infections.^11^

Medication errors (MEs) are established safety concerns in all individuals consuming medicines,^12^ defined as *failures in the treatment process that have the potential to cause harm to the patient*.^13^ Harm caused by MEs includes prolonged hospital admission, hospital readmission or increased mortality.^12,14^ MEs can happen at any stage of the medication use process, including prescribing, documenting, transcribing, dispensing, administration, and monitoring.^15^ ART related MEs may result in toxicity, failure of therapy, and the development of viral resistance, which must be prevented or corrected swiftly.^14,16^

MEs are described as the third global patient safety challenge by the World Health Organization (WHO).^12,17^ Studies conducted in the United States reported a prevalence of 25.8% – 72% MEs amongst hospitalised PLHIV.^18^

Only five ME studies occurred in SA between 2004 and 2009, none in PLHIV,^19^ restricting knowledge regarding the extent of the problem. The increased risk for hospitalised patients is further elevated due to transitioning between primary healthcare and secondary or tertiary hospitals.^16^

Pharmacists are experts in pharmacology, pharmacokinetic- and pharmacodynamic principles, understanding standard treatment plans, and identifying drug interactions and potential MEs through prescription evaluation. They can identify challenges in patients with new diseases requiring treatment.^16^ A systematic review and meta-analysis on MEs highlighted the benefits of pharmacist involvement in patient care, improving medication use and reducing medication-related costs. This review concluded that pharmacist involvement is essential to reducing MEs, regardless of the population involved.^20^

Schellack et al.^21^ reported that more than 50% of MEs, including 72% of potentially dangerous errors, may be prevented when pharmacists monitor medication orders. Additionally, medication reconciliation may improve communication between pharmacists and other healthcare workers, potentially preventing 47.4% of errors.^21^

Pharmacist-led services to minimise MEs have demonstrated reductions in MEs during hospitalisations. However, the role of the pharmacists in PLHIV is not clearly described in SA. Therefore, the purpose of this study was to evaluate the prevalence and type of MEs occurring in PLHIV admitted to a tertiary hospital in the Western Cape, and to describe pharmacist recommendations from this evaluation.

## Research methods and design

### Study design

A prospective observational study was conducted over a period of 14 weeks, reviewing patient files to determine the prevalence and type of related MEs occurring in PLHIV at the study site. The study clinical pharmacist identified MEs, recommended appropriate actions to the prescriber, and documented resolution of the MEs.

### Setting

This study was conducted at a 945-bed tertiary academic hospital in the Western Cape, SA, where the researcher is employed. It included patients from four general Internal Medicine wards.

### Study population and sampling strategy

The study population included all patients who were diagnosed with HIV before, at the time of, and during hospital admission.

Homogeneous purposive sampling, as described by Palinkas et al.,^22^ was used to select study participants, which allowed the researchers to review only PLHIV. This sampling method was conducive to reviewing only data required to determine the prevalence and type of MEs observed in hospitalised PLHIV.^22^

The Raosoft^®^ sample size calculator, an online application developer for web surveys, was used to identify a sample size of *n* = 180 participants, with a 95% confidence level, a 3% margin of error, a response distribution of 50% and a PLHIV population of 280 in the preselected wards.

### Data collection

A data collection instrument (DCI) was designed and adapted from previous studies by Commers et al., Guo et al., and Pittman et al.,^7,8,18^ to ensure that the required data were collected. Information collected on the DCI included demographic data, including gender, age, weight and duration of admission, the patient’s medical history, reasons for the current admission, and prescribed medication. Furthermore, identified MEs were listed, together with recommended actions to resolve errors.

Up-to-date^®^, EMGuidance™, Essential Medicine List Antiretroviral interaction table, Medscape™ drug interaction checker and the South African Medicines Formulary were used in order to identify and verify MEs and drug interactions. The pharmacist’s involvement was determined by quantifying the number of MEs corrected by the pharmacist’s interventions or recommendations.

### Data analysis

The data were analysed using an Excel™ spreadsheet and the IBM Statistical Package for Social Science version 28 (IBM Corp., Armonk, New York, United States). Descriptive summaries were generated for both demographic and hospital admission data. Admission data were used to summarise clinical information and evaluate study outcomes. MEs identified were quantified using frequencies and percentages, and the time to resolve MEs was determined.

### Ethical considerations

The study was approved by the Sefako Makgatho Health Sciences University Research and Ethics committee (SMUREC) (SMUREC/P/92/2021: PG), the Western Cape Department of Health as well as the Chief Operations Officer of the hospital. The data collected are for research purposes exclusively and patients’ written informed consent was obtained prior to inclusion. Confidentiality was maintained by using study codes for participants. Study-related documents are stored on a personal computer with password protection for 5 years.

## Results

### Demographic data

A total of 280 hospitalised patients fulfilled the inclusion criteria for this study. However, *n* = 180 were recruited, as 100 patients could not be recruited due to their inability to provide consent for various reasons, including their medical condition and unresponsiveness.

Files of the study participants (*n* = 180) were reviewed for ART regimens and co-morbidities. Most patient files were reviewed more than once, from admission and throughout their admission to the preselected general wards, resulting in *N* = 453 reviews during the study period. Table 1 provides an overview of the demographic characteristics of the study population.

**TABLE 1:** Population data.

Variable	Category	Mean ± s.d.	Range (years)	*n*	%
Study population	280 fulfilled inclusion criteria 100 did not provide consent	-	-	180	-
Gender (*N* = 180)	Male	-	-	75	41.6
	Female	-	-	105	58.3
Age (years)	-	40.09 ± 19.75	18–97	-	-
Provision of consent	Patient’s consent (self)	-	-	158	87.8
	Doctor’s consent on behalf of patient	-	-	22	12.2

s.d., standard deviation.

### Co-morbidities

**FIGURE 1:**
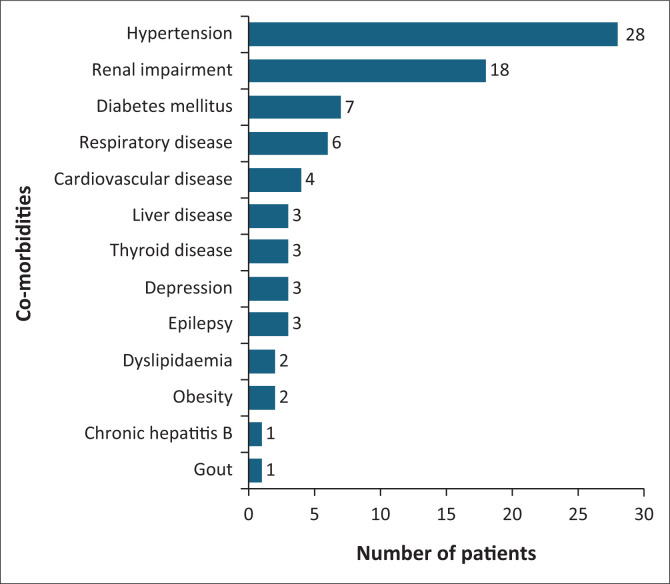
Co-morbidities observed in study participants.

This study included *n* = 180 hospitalised patients during the data collection period. The patients’ medical history included polysubstance abuse for 25 (13.9%) participants, 69 (38.3%) were previously diagnosed with tuberculosis (TB), five (2.8%) had a previous cerebrovascular incident and seven (3.9%) had coronavirus disease 2019 (COVID-19). The study did not quantify the COVID-19 vaccination of participants. Co-morbidities were identified in 45 (25%) participants, with 22 having one co-morbidity, 20 having two or more co-morbidities, and three having four co-morbidities. The most common co-morbidities observed are represented in Figure 1.

### Opportunistic infections

**FIGURE 2:**
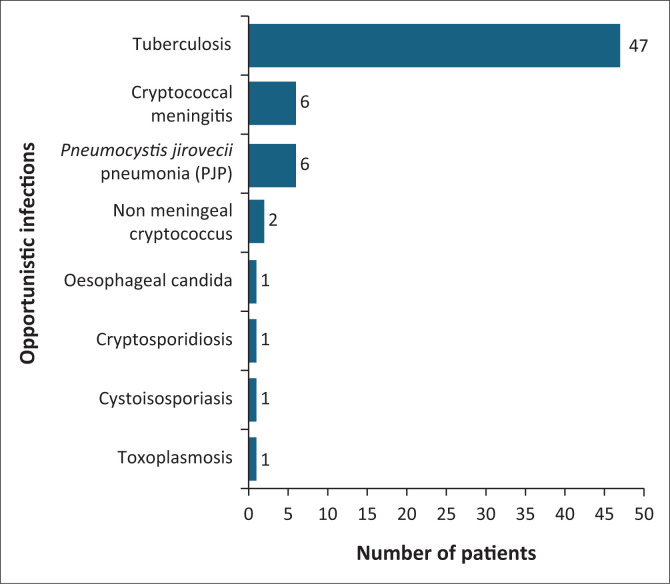
Opportunistic infections observed in study participants.

Opportunistic infections (OIs) were identified in 62 (34.4%) participants; three participants experienced two OIs simultaneously. The most common OI observed was TB (26.1%). OIs and frequencies are represented in Figure 2.

### Medication errors observed in hospitalised people living with HIV and AIDS

MEs were observed during 316 (69.8%) reviews, and 466 MEs were identified and documented, with an average of 1.03 errors per review (Table 2). Table 2 presents the time taken to resolve MEs after a recommendation to the prescriber.

**TABLE 2:** Categories of medication errors observed.

Error type	Description	Example	Number of errors (*N* = 466)	Percentage of errors (*N* = 466)	Time to resolution		
			*n*	%	< 24 h	> 24 h	Not resolved
Incorrect medication reconciliation from history	Incorrect medication reconciliation from history	ART history: TLD FDC tablet, however TEE FDC tablet prescribed on this admission.	19	4.0	15	2	2
Prescription omission	Medication omitted	Previous prescription included CTX and TLD FDC; however, both omitted from this prescription.	17	3.6	12	2	3
Duplication of therapy	Additional medication in same class administered	Carvedilol prescribed twice (initial 3.125 mg BD changed to 6.25 mg BD). First prescription not stopped and administered both doses.	10	2.1	8	0	2
Missed doses	Medication not administered	Patient missed doses of the following agents including RIF, INH, ETH, PZA DTG, heparin SC, paracetamol.	265	56.9	163	31	71
**Incorrect dosing**			103	22.1	47	23	33
Under	Incorrect medication dosing (from guidelines); appropriate dose adjustment according to body weight, renal function	Prescribed LOP/RIT FDC 2 tablets BD, with rifampicin. Unboosted LOP/RIT.	37	7.9	-	-	-
Over		Patient prescribed bedaquiline 400 mg D, longer than initial 2 weeks during therapy induction.	55	11.8	-	-	-
Both		Prescribed ABC 300 mg D, 3TC 150 mg D in presence of eGFR 1 mL/min. Recommended dosing guidelines in adults are ABC 600 mg D and 3TC 25 mg D.	11	2.4	-	-	-
**Administration**							
Frequency of administration	Medication administered at the incorrect frequency	-	2	0.4	1	1	0
Duration more than prescribed	Medication administered longer than prescribed	Prescribed furosemide for 3 days, administered for an additional 2 days.	15	3.2	7	8	0
Drug-drug interactions	Drug interaction between two or more prescribed medication	Prescribed TLD with Rifafour^®^. No booster dose of DTG.	18	3.9	13	1	4
Other	Any other ME not previously indicated	Continued taking TEE while not prescribed. The patient developed DILI and the notes indicated to stop ART.	17	3.7	6	4	7

ART, antiretroviral therapy; TLD, tenofovir, lamivudine and dolutegravir; FDC, fixed-dose combinations; TEE, tenofovir, emticitrabine, efavirenz; CTX, co-trimoxazole; BD, twice a day; RIF, rifampicin; INH, isoniazide; ETH, ethambutol; PZA, pyrazinamide; DTG, dolutegravir; SC, sub-cutaneous; LOP/RIT, lopinavir/ritonavir; D, daily; ABC, abacavir; 3TC, lamivudine; eGFR, estimated Glomerular filtration rate; DILI, drug-induced liver injury; ME, medication error.

Missed doses were observed in 265 reviews (*N* = 453, 56.9%), yielding 406 medicines that had missed doses. The top five medication classes identified with missed doses were analgesics (*n* = 127; 25%), antiretroviral agents (*n* = 53; 10.5%), supplements (*n* = 50; 9.9%), antibiotics (*n* = 44; 8.7%), and antiemetics (*n* = 27; 5.3%). Explanations for why medication doses were missed were only documented for 35 (13.2%) cases, for example omitting antithrombotic treatment because of high INR.

The incorrect dosing regimen was observed in 103 reviews (*N* = 453, 22.7%), identifying 136 inappropriate doses (1.3 dosing errors per review). A single error was observed in 73 reviews and one review had five dosing errors. The top medicine groups involving dosing errors included antimycobacterial agents (*n* = 42; 30.8%) and antiretroviral agents (*n* = 26; 19.1%). The most common individual agents dosed incorrectly included Rifafour^®^ (*n* = 26; 19.1%), enoxaparin (*n* = 16; 11.8%), lamivudine (*n* = 15; 11%), fluconazole (*n* = 10; 7.4%) and co-trimoxazole (*n* = 6; 4.4%).

Drug-drug interactions (DIs) were the only interaction observed in this study and accounted for 18 MEs (*N* = 453; 4%). Unboosted tenofovir, lamivudine and dolutegravir (TLD) prescribed simultaneously with rifampicin was observed nine times. Seven of these errors were resolved in less than 24 h. Drug-supplement interactions were attributed to seven interactions, for example Citrosoda^®^ (sodium citrate) and isoniazid (INH), thereby reducing INH absorption. Five of these errors were resolved more than 24 h later. Unboosted lopinavir/ritonavir prescribed with rifampicin was observed twice. One was resolved in less than 24 h and one was resolved more than 24 h later.

### Medication errors observed change at the point of care

**FIGURE 3:**
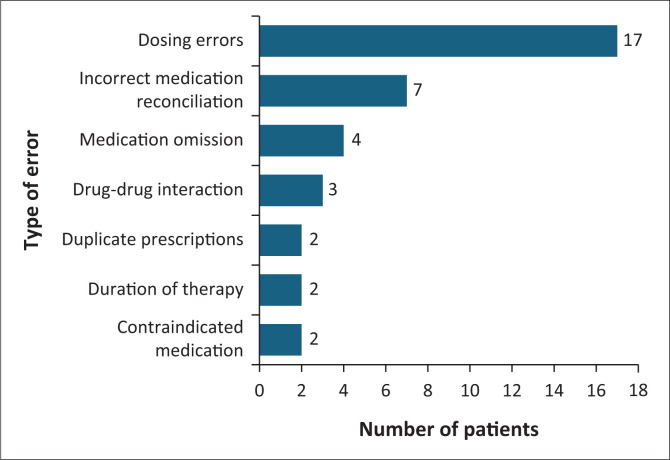
Medication errors observed on transition of care prescriptions.

Medication reconciliation was performed for 171 (95%) patients during the transition of care, eight (0.04%) demised and one participant discharged themselves against medical advice. Medication was issued to 143 (79%) patients during the transition of care. Medication errors were observed in 37 of the 143 prescriptions. The types of MEs observed included dosing errors, incorrect medication reconciliation, medication omission, DIs, duplicate prescriptions, duration of therapy and contraindicated medication (Figure 3). Seventeen (*n* = 17; 46%) MEs were resolved before patients’ transition of care.

## Discussion

Co-morbidities were identified in a quarter of participants. Godongwana et al.^23^ stated that approximately one-third of virally suppressed South African PLHIV have at least one co-morbidity.^23^ The most common co-morbidities observed in the present study included hypertension, both acute and chronic renal impairment, and diabetes mellitus. The general prevalence of hypertension in adults 18 years and above is around 27%.^10^ PLHIV present with similar co-morbidities, but also cardiovascular, respiratory and hepatic diseases in addition.^10^ Brennan et al.,^24^ however, found a 22% prevalence of hypertension at ART initiation in SA, which is less than the current study’s findings of 28%, and could be attributed to only including ART-naïve, non-pregnant PLHIV adults who were newly initiated on standard first lines.^24^ Vachiat et al.^25^ found a 14.8% prevalence of PLHIV presenting with renal failure to a tertiary hospital in Johannesburg,^25^ while 9% prevalence of diabetes mellitus in PLHIV in KwaZulu-Natal was found.^26^ Both were higher than the results from this study.

The most common OIs observed during this study were TB, Cryptococcal meningitis (CCM) and *Pneumocystis jirovecii* pneumonia (PJP). SA has one of the highest TB burdens globally.^27^ This study population had 26.1% of participants diagnosed with active TB, which is in line with the first national TB prevalence survey, which reported that 28% of PLHIV in SA were co-infected with TB.^27^

CCM was diagnosed in 3.3% of participants, which was lower than the 6.5% found in a meta-analysis of pooled global prevalence of cryptococcal antigenaemia.^28^ Sonderup et al.^29^ described the negative consequences of COVID-19 on non-COVID general medical and routine public health services, finding that general medicine services were scaled back to ensure resources were redirected to the COVID-19 ward.^29^ This could explain the lower-than-expected CCM prevalence observed in the present study.

PJP was diagnosed in 3.3% of study participants, which is much lower than the 22.4% prevalence in sub-Saharan Africa.^30^ The lower-than-expected prevalence of PJP in this study could be explained by Maartens et al.,^30^ who describes PJP as difficult to diagnose in low- to middle-income countries due to limited resources such as bronchoscopy facilities, sophisticated imaging and microbiological identification, and co-infection with TB.^30^ The increased roll-out and expansion of ART accessibility, as well as the regular use of chemoprophylaxis for OIs, may further justify the lower-than-expected PJP prevalence.^31^

People living with HIV/AIDS are at greater risk for MEs due to complex medication regimens, polypharmacy, DIs, increased hospitalisations for other co-morbid conditions, other infections, and knowledge limitations of healthcare providers.^7,8,9^ MEs were observed in 69.8% of the reviews in the current study, and 94.4% of participants experienced MEs during their hospital admission. Gou et al.^8^ indicated that 84% of hospitalised PLHIV experience an ART-related error.^8^ The high prevalence of MEs observed in the study can be attributed to the fact that this study evaluated all MEs experienced during admission, and not only ART-related MEs. In contrast, Otwombe et al.,^13^ found very low reported MEs in their study tracking MEs in the Central Chronic Medicine Dispensing and Distribution (CCMDD) programme in SA. Their conclusion was that the CCMDD programme was very well controlled.

The most common MEs observed in this study include missed doses, incorrect medication dosing and DIs. Similarly, although in a smaller population, inappropriate ARV regimens and dosing errors were the most prevalent MEs observed in a study in the United States.^32^

Carcelero et al.^6^ reported that 15% of their study population experienced omitted ART medication doses.^6^ Green et al.^33^ found that 20% of prescription charts reviewed experienced omitted medication during admission and documented all medication not administered within the first 48 h of admission.^33^ Adherence to prescribed medication is described as taking at least 95% of treatment as prescribed.^34^ Missed ART doses accounted for 10.5% of missed doses, which indicates poor ART adherence with numerous negative consequences including treatment failure, viral mutations, and the development of resistance.^34,35^ Decano et al.^35^ found that their sample population had an overall adherence rate of 91%; 40% experienced at least one missed dose and missed doses were more associated with multi-tablet regimens (89%) than single-tablet regimens (11%). In this study, however, all patients that had missed doses were on fixed-dose combination (FDC) antiretroviral agents and missed doses because of other reasons, such as failure to prescribe, or unknown regimens when admitted. While Decano et al.^35^ only evaluated ART, their investigations still support the findings of this study, which evaluated all the study participants’ medication administration.^35^ Hareru et al.^36^ conducted a study in Ethiopia and found 60.1% non-adherence to prescribed antibiotics. This is much higher when compared to the 8.7% non-adherence observed in this study and the international average of 50%.^36^ The huge difference is attributed to their study only evaluating non-adherence to antibiotics in Southern Ethiopia.

Gou et al.^8^ and Commers et al.^18^ found that 13.8% and 14.6% of MEs observed in their respective studies were attributed to incorrect medication dosing of ART.^18^ Otwombe et al.^13^ found fewer MEs in the CCMDD programme. The most prevalent ME was incorrect dosing, supporting the study’s observation that 19.1% of dosing errors were observed in ARTs.

Even PLHIV during early HIV-1 infection on ART are up to five times more likely to develop active TB, and those with advanced HIV are 20 times more likely to develop active disease.^37^ Considering this and the findings of Van Der Walt et al.^27^ on the prevalence of TB co-infected in PLHIV in SA (28% in 2018), the study found that 30.8% of dosing errors observed in anti-mycobacterial agents is plausible.^27^ This is also evident when nine of the DIs observed related to booster doses of dolutegravir (DTG) that were not prescribed, and two of the DIs observed related to booster doses of lopinavir/ritonavir that were not prescribed, together with rifampicin-based TB therapy. Most of the research studies focus on ART-related MEs, whereas the present study investigated MEs occurring in all classes of medication prescribed in PLHIV.

Medication errors attributed to DIs accounted for 3.9%. Unboosted TLD prescribed with rifampicin-based TB therapy accounted for 50% while unboosted lopinavir/ritonavir prescribed with rifampicin-based TB therapy accounted for 11.1% of all DIs. The findings of Van Der Walt et al.,^27^ on the prevalence of TB co-infection PLHIV in SA being 28%, support the high number of interactions with TB medication in the present study. Yehia et al.,^38^ Chiampas et al.^39^ and Gou et al.^8^ found 13.0%, 9.0% and 10.2% of their study’s MEs were attributed to DIs.^8,38,39^ However, protease inhibitors were involved in most of the DIs observed in Yehia et al.,^38^ Chiampas et al.^39^ and Gou et al.^8^ (100%, 97% and 79%, respectively). The use of TLD as first-line ART was introduced in 2019 as part of the updated ART Clinical Guidelines for the Management of HIV in Adults, Pregnancy, Adolescents, Children, Infants and Neonates.^40^ Changes in the standard treatment guidelines, the high prevalence of TB co-infection, and the information gaps regarding the need for a booster dose of DTG with TLD when patients are on a rifampicin-based TB regimen, all contribute to the occurrence of this drug interaction.

The pharmacist made recommendations to prescribers and nursing sisters at the point of care, which resolved more than 50% of MEs in less than 24 h. This highlights the significant role pharmacists can play in resolving MEs rapidly and ultimately preventing treatment failure, ADEs and improving patient outcomes. Yehia et al.^38^ reported that 76% of documented MEs were corrected within 48 h. The rapid correction of MEs was attributed to two clinical pharmacists specialising in infectious diseases, who reviewed all medication orders.^38^ This study’s results differed from Yehia et al.,^38^ due to dedicated infectious disease clinical pharmacists using computerised provider order entry systems.

Nonetheless, by addressing MEs during hospital stays, a pharmacist can facilitate a hospitalised patient’s smooth transition to a point of care smoothly, limiting MEs.^16^

### Limitations

Data collection was restricted to four preselected general medical wards, limiting the generalisation of the results to the general population, or even all hospitalised patients. Furthermore, the outcomes of interventions were only quantified, and the clinical and cost implications were not studied.

### Recommendations

Active participation of pharmacists in patient reviews during hospitalisation will assist with the identification of MEs as well as other problems identified during the hospitalisation or at the transition of care. The significance of these DIs must be communicated to prescribers and nursing staff at large, to ensure patients do not experience treatment failure because of negligence to prescribe booster DTG, or failure to increase doses of lopinavir/ritonavir when TB co-infection is also being treated.

Pharmacists at ward level can assist in the rapid resolution of medication-related errors and queries, with a possible reduction in patient hospitalisation and healthcare costs.

## Conclusion

MEs in hospitalised patients are common. The findings of this study provide information regarding the extent of MEs that occur in hospitalised PLHIV. This information and understanding of the extent of the problem in HIV-positive patients can empower healthcare workers to develop targeted systems and interventions to address and to prevent unnecessary MEs or omission in this vulnerable population. The study highlights the value of the clinical pharmacist in reducing the number of MEs significantly. The reduction of MEs throughout a patient’s hospitalisation, through intervention and quality improvement projects, improve patient care to protect PLHIV.
